# Extract of *Antrodia camphorata* Exerts Neuroprotection against Embolic Stroke in Rats without Causing the Risk of Hemorrhagic Incidence

**DOI:** 10.1155/2014/686109

**Published:** 2014-07-21

**Authors:** Ye-Ming Lee, Chiu-Yun Chang, Ting-Lin Yen, Pitchairaj Geraldine, Chang-Chou Lan, Joen-Rong Sheu, Jie-Jen Lee

**Affiliations:** ^1^Department of Surgery, Hsinchu Mackay Memorial Hospital, Hsinchu 300, Taiwan; ^2^Department of Nursing, Mackay Medicine, Nursing and Management College, Taipei 112, Taiwan; ^3^Graduate Institute of Medical Sciences, College of Medicine, Taipei Medical University, 250 Wu-Hsing Street, Taipei 110, Taiwan; ^4^Department of Anatomy, School of Medicine, Taipei Medical University, Taipei 110, Taiwan; ^5^Department of Animal Science, School of Life Sciences, Tiruchirappalli, Tamil Nadu 620 024, India; ^6^Sheen Chain Biotechnology, Co., Ltd., Taipei 115, Taiwan; ^7^Mackay Junior College of Medicine, Nursing, and Management, Mackay Memorial Hospital, Taipei 112, Taiwan; ^8^Department of Surgery, Mackay Memorial Hospital, No. 92, Section 2, Zhongshan N. Road, Taipei 104, Taiwan

## Abstract

In this study, the neuroprotective effect of an extract of *Antrodia camphorata* (*A. camphorata*), a fungus commonly used in Chinese folk medicine for treatment of viral hepatitis and cancer, alone or in combination with aspirin was investigated in a rat embolic stroke model. An ischemic stroke was induced in rats by a selective occlusion of the middle cerebral artery (MCA) with whole blood clots and then orally treated with *A. camphorata* (0.25 and 0.75 g/kg/day) alone and combined with aspirin (5 mg/kg/day). Sixty days later, the brains were removed, sectioned, and stained with triphenyltetrazolium chloride and analysed by a commercial image processing software program. Brain infarct volume, neurobehavioral score, cerebral blood perfusion, and subarachnoid and intracerebral hemorrhage incidence were perceived. In addition, potential bleeding side effect of the combinative therapy was assessed by measuring hemoglobin (Hb) content during intracerebral hemorrhage and gastric bleeding, prothrombin time (PT), and occlusion time (OT) after oral administration. Posttreatment with high dose *A. camphorata* significantly reduced infarct volume and improved neurobehavioral score (*P* < 0.05). Since *A. camphorata* alone or with aspirin did not alter the Hb level, this treatment is safe and does not cause hemorrhagic incident. Remarkably, the combination of *A. camphorata* and aspirin did not show a significant effect on the bleeding time, PT and OT increase suggesting that *A. camphorata* may have the neuroprotective effect without the prolongation of bleeding time or coagulation time. From these observations, we suggest that combinative therapy of *A. camphorata* and aspirin might offer enhanced neuroprotective efficacies without increasing side effects.

## 1. Introduction

Stroke is the second most leading cause of death and the first cause of major adult disability in the world [1, 2]. Among the stroke patients, 85–90% of the cases are ischaemic stroke with a major (75–80%) cause of cerebral arterial thrombosis, and majorities of ischaemic incidents ensue as a result of occlusion of the middle cerebral artery [3, 4]. Mortality and serious disability result if patients are not treated successfully within 30–90 min after onset of symptoms. Aspirin is the most widely used drug for the secondary prevention of thrombotic events, due to its antiplatelet action. It is effective for the prevention of secondary stroke and has been used in up to 89% of patients in China. Nevertheless, while recurrent stroke is controlled by this treatment, the incidence of cerebral haemorrhage and other bleeding events is higher in Asian countries than in high-income countries [5]. Hence, combinations of antiplatelet drugs that act on different pathways have been used to overcome the risk of haemorrhage. These compounds have been shown to be used efficiently and safely in patients with unstable coronary heart disease. Recent studies have revealed that the combination of two antiplatelet drugs is disastrous to recover stroke prevention rates owing to the increased risk of bleeding events associated with their long-term use [6, 7]. Bleeding due to antiplatelet drugs is a vital clinical issue in primary and secondary stroke prevention, mostly in the Chinese population, which has a higher incidence of cerebral haemorrhage than other indigenous groups [8].

*A. camphorata* is one of the complementary alternative medicines. It is a parasitic fungus that only grows on the inner heartwood wall of* Cinnamomum kanehirai* Hay (Lauraceae).* A. camphorata* is usually used in Taiwanese folk medicine for abdominal pain, chemical intoxication, diarrhea, hypertension, itchy skin, and hepatoma [9]. Recent studies have demonstrated that* A. camphorata* induces significant apoptosis of human promyelocytic leukemia (HL-60) cells [10]. Another study proved that* A. camphorata* extracts may be used as an adjuvant antitumor agent for human hepatoma cells, which are resistant to most other antitumor agents. Our previous study demonstrated that* A. camphorata* owns effective protection against carbon tetrachloride- (CCl_4_-) induced hepatic injury in vivo, by mediating antioxidative and free radical scavenging activities [11], and it has shown to reduce H_2_O_2_-induced lipid peroxidation and upregulating hepatic glutathione-dependent enzymes for protecting the rat liver from the CCl_4_-induced damage [12].

The embolic model has been used previously in different experiments to induce experimental stroke in rodents [13], as this model mimics human stroke and is more relevant to the pathophysiological situation in patients. Besides, different sizes of lesion could be created in this model by injection of various volumes of clot into the middle cerebral artery [14]. Therefore, we investigated whether a combination of* A. camphorata* with the lowest effective dose of aspirin may provide more neuroprotection during the embolic model of stroke in rats without increasing the potential bleeding side effect and different hemorrhagic incidents.

## 2. Materials and Methods

### 2.1. Plant Material

The crude extracts of* A. camphorata* were offered by Well Shine Biotechnology Development Co., Pvt. Ltd., Taipei, Taiwan.

### 2.2. Animals

Male Wistar rats weighing 250–300 g were used to assess the effects of* A. camphorata* given alone or in combination with aspirin on MCAO-induced brain damage. Animal care and the general protocols for animal use were approved by the Institutional Animal Care and Use Committee (IACUC) of Taipei Medical University (no. LAC-101-0239). Before undergoing the experimental procedures, all animals were clinically normal, free of apparent infection or inflammation and showed no neurological deficits.

### 2.3. Middle Cerebral Artery Occlusion- (MCAO-) Induced Ischemia

Rats were subjected to MCAO-induced ischemia by administration of an autologous blood clot as described in our previous study [15]. Briefly, arterial blood (0.6 mL) was withdrawn from a femoral catheter in a 1 mL syringe. The blood was immediately injected into PE-10 tubes. The tubes were kept at 4°C for 22 h, and the thread-like clots were removed and placed in a saline-filled dish. The clots were then washed to remove blood cells. Washed portions of the clots were transferred to fresh dishes, and the washing process was repeated until the saline remained clear. These clot sections were cut into 30 mm long fragments and then drawn up with the saline solution into a PE-10 catheter.

On the day of surgery, animals were anesthetized with a mixture of 75% air and 25% O_2_ gases containing 3% isoflurane. The common carotid artery (CCA) was identified, and approximately 1 cm of the external carotid artery (ECA) was ligated and cut. Subsequently, the pterygopalatine artery (PA) was clamped with a 10 mm microaneurysm clamp, and the CCA was similarly clamped before the carotid bifurcation. The internal carotid artery (ICA) was then clamped between the carotid bifurcation and the PA. Next, the PE-50 catheter containing the clot was introduced approximately 5 mm into the previously cut ECA and tied in place with sutures. The ICA clamp was removed, and the clot was flushed into the ICA over a period of approximately 5 s. The PA clamp was removed, and the rat was left in this condition for 1 h.

### 2.4. Experimental Design

In this study, at 1 hr after MCA occlusion, rats were randomly separated into six groups: (1) a sham-operated group; (2) a group orally treated with an isovolumetric solvent (distilled water) for 60 days, followed by thromboembolic occlusion; (3 and 4) groups orally treated with* A. camphorata* (0.25 and 0.75 g/kg/day) alone for 60 days, followed by thromboembolic occlusion, respectively; and (5 and 6) groups treated with* A. camphorata* (0.25 and 0.75 g/kg/day) and aspirin (5 mg/kg/day), followed by thromboembolic occlusion, respectively. An observer blinded to the identity of the groups assessed the neurological deficits after reperfusion (before being euthanized) by forelimb akinesia (also called the postural tail-hang) test.

### 2.5. Quantification of Brain Infarct Volume

Rats were euthanized by decapitation after 24 h of reperfusion. The brains were cut into 2 mm coronal slices starting 1 mm from the frontal pole. Each stained brain (2% 2,3,5-triphenyltetrazolium; TTC) slice was drawn using a computerized image analyzer (Image-Pro Plus). The calculated infarct areas were then compiled to obtain the infarct volume (mm^3^) per brain. Infarct volumes were expressed as a percentage of the contralateral hemisphere volume using the formula (the area of the intact contralateral [left] hemisphere—the area of the intact region of the ipsilateral [right] hemisphere) to compensate for edema formation in the ipsilateral hemisphere [15].

### 2.6. Neurological Functional Tests

Sensorimotor integrity was measured in rats at 1 and 24 h after MCAO by an investigator blind to the experimental groups to assess the neurobehavior [16]. Scoring was as follows: 0: no observable deficit, 1: forelimb flexion, 2: forelimb flexion plus decreased resistance to lateral push, 3: unidirectional circling, and 4: unidirectional circling plus decreased level of consciousness.

### 2.7. Evaluation of Hemorrhagic Incidence

As previously published, hemoglobin content in the ischemic hemisphere and gastric luminal fluid was determined as an index of intracerebral hemorrhage and gastric bleeding using a colorimetric method as described by a kit (Haemoglobin, HG1539) purchased from Randox Lab Ltd., UK. The hemoglobin content in ischemic hemisphere and gastric luminal fluid was determined by using the optical density readings obtained from the known hemoglobin standards and reported in g/dL.

### 2.8. Grading System for Subarachnoid Hemorrhage (SAH)

**Figure 1:**
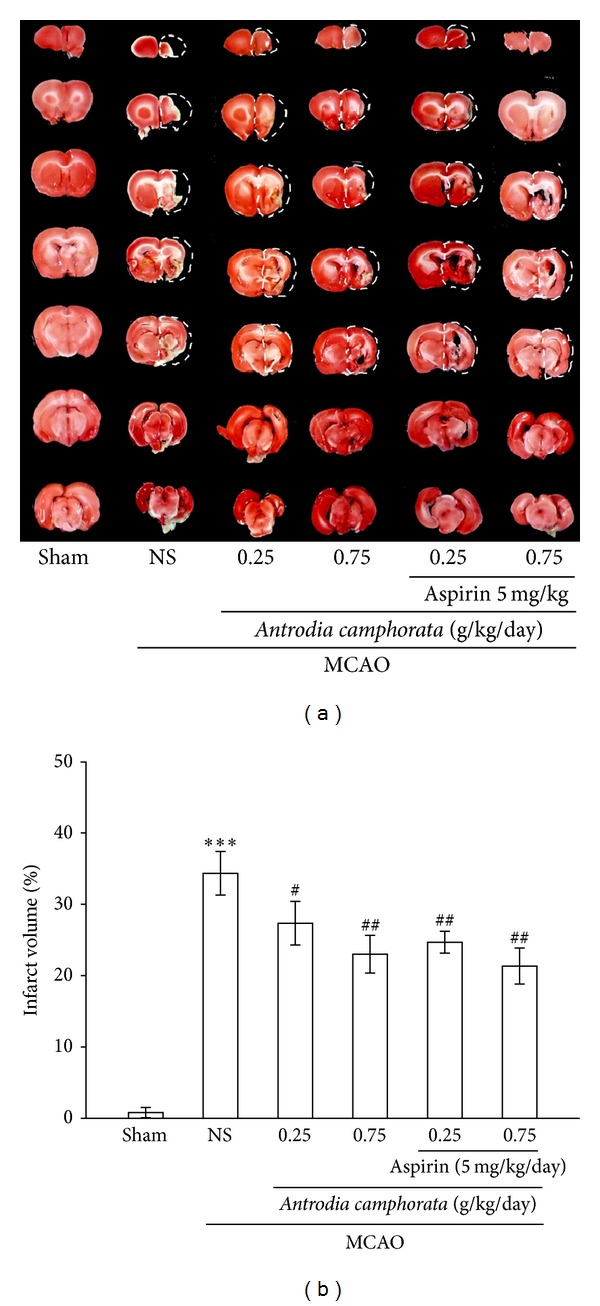
(a) Combination treatment of the extract of* A. camphorata* with aspirin against thromboembolic stroke in rats. Coronal sections of 2,3,5-triphenyltetrazolium- (TTC-) stained brains after thromboembolic occlusion-reperfusion rats were observed in a sham-operated group (sham), a group orally treated with an isovolumetric solvent (distilled water) for 60 days, followed by thromboembolic occlusion (MCAO group), groups orally treated with* A. camphorata* for 60 days alone, followed by thromboembolic occlusion, and groups orally treated with* A. camphorata* (0.25 and 0.75 g/kg/day) combined with aspirin (5 mg/kg/day) for 60 days, followed by thromboembolic occlusion as described in “Section 2.” The results are representative examples of three similar experiments; (b) densitometric analysis of the combination treatment of the extract of* A. camphorata* with aspirin against thromboembolic stroke in rats. Data are presented as the infarct volume for each animal in the group as well as the means ± S.E.M. (*n* = 5). ***P < 0.001 compared to the sham-operated group, ^#^ *P* < 0.05 and ^##^ *P* < 0.01 compared to the MCAO group.

The rats were sacrificed under deep anesthesia at 60 days after surgery and the brains were removed rapidly. High resolution pictures of the base of the brain depicting the circle of Willis and basilar arteries were taken. In the photographs, the basal cistern was shown in Figure 1(a). This grading was done by a blinded observer.

### 2.9. Measurement of Prothrombin Time (PT)

Measurement of prothrombin time (PT) was performed by using a kit (Instrumentation Laboratory, Milano, Italy) as described [17]. Briefly, just before ligation to form a snare in the rat Wessler model, arterial blood samples (3 mL) were withdrawn; then the artery was ligated immediately; 0.9 mL of each blood sample was transferred into a 0.109 M trisodium citrate solution (1 : 9, v/v) and then centrifuged at 2000 ×g for 10 min to obtain plasma. 100 *μ*L plasma was mixed with 50 *μ*L of cephalin in a process plate, and the coagulation was started by the addition of CaCl_2_ (1 mM), 100 *μ*L of thromboplastin, and 100 *μ*L of bovine thrombin into the 100 *μ*L of incubated plasma for PT assay.

### 2.10. Measurement of Occlusion Time (OT)

As described previously [15], mice were anesthetized, and an external jugular vein was cannulated with PE-10 so that dye and medication could be administered by an intravenous (i.v.) bolus. A segment of the small intestine was placed onto a transparent culture dish for microscopic observation. Venules (30 to 40 *μ*m) were selected for irradiation to produce a microthrombus. Using the epi-illumination system, light from a 100 W mercury lamp was passed through a B-2A filter (Nikon, Tokyo, Japan) with a DM 510 dichromic mirror (Nikon). Wavelengths below 520 nm had been eliminated from the filtered light, which was used to irradiate a microvessel; the area of irradiation was approximately 100 *μ*m in diameter on the focal plane. A dose of 0.75 g/kg/dayof* A. camphorata* was administered 3 min after fluorescein sodium administration. Five minutes after administration of the fluorescein sodium, irradiation by filtered light and the video timer were simultaneously begun, and occlusion time was observed on a television monitor. The time lapse for inducing thrombus formation leading to the cessation of blood flow was measured.

### 2.11. Data Analysis

Experimental results are expressed as the mean ± S.E.M. and are accompanied by the number of observations. The experiments were assessed by the method of analysis of variance (ANOVA). If this analysis indicated significant differences among the group means, then each group was compared using the Newman-Keuls method. A *P* value of <0.05 was considered statistically significant.

## 3. Results

### 3.1. *A. camphorata* and Aspirin Combination Therapy Reduces Infarct Volume

The cerebral infarction was examined using 2 mm thick slices of the cerebrum 24 h after MCAO reperfusion in rats through TTC staining. Figure 1(a) shows typical photographs of coronal sections of sham-operated group, MCAO-treated group,* A. camphorata*-alone-treated groups (0.25 and 0.75 g/kg/day), and* A. camphorata* + aspirin (5 mg/kg/day) treated groups prior to the ischemic insult. At 24 h after MCAO, a high dose of* A. camphorata* treatment alone or combination treatment with aspirin reduced the infarct volume, both of which were more significantly reduced than the volume of MCAO-induced untreated group (Figure 1(b)). In addition, while even a high dose (0.75 g/kg/day) of* A. camphorata* combined with aspirin (5 mg/kg/day) did not show any significant effect on reducing the infarct volumes (Figure 1(b)), as it is found to be almost comparable to that of high dose* A. camphorata* treatment alone, the aspirin has been removed in the following studies.

### 3.2. *A. camphorata* Treatment Improves Neurological Outcome and Blood Perfusion Deficit

**Figure 2:**
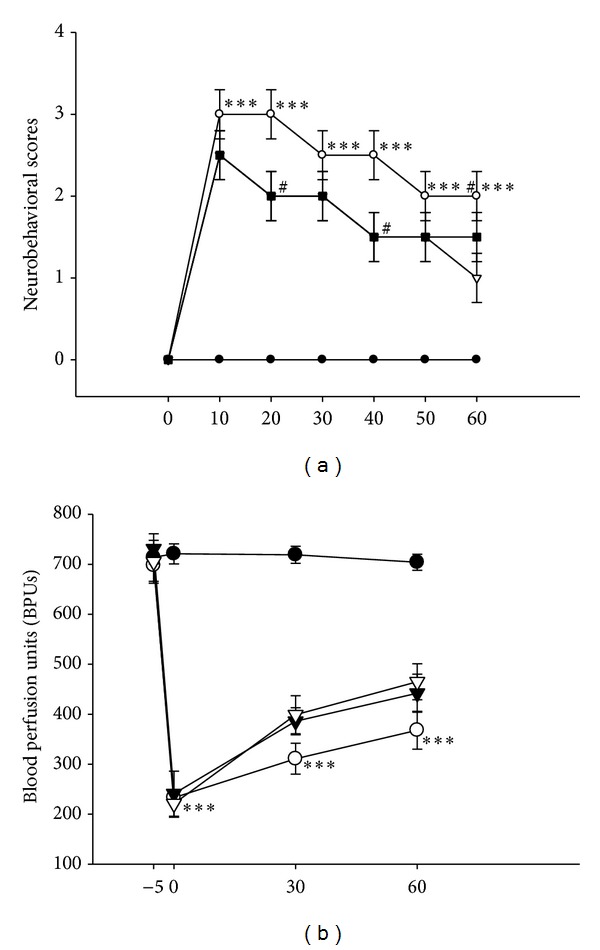
Effects of the extract of* A. camphorata* on (a) neurobehavioral deficits and (b) blood perfusion unit (BPU) in thromboembolic stroke-induced rats. (a) shows neurobehavioral deficits in 4 experimental groups; (b) shows BPU of solvent and* A. camphorata* or the aspirin treated rats were measured by laser Doppler flowmeter in the MCAO-supplied cortex. Data are presented as the means ± S.E.M. of three similar experiments.***P < 0.001 compared to the sham-operated group; ^#^ *P* < 0.05 compared to the MCAO group.

Variations of neurological deficits scores in different groups are shown in Figure 2(a). Before MCAO, the neurological score was zero in all animals. After MCAO, high-grade neurological deficits (*P* < 0.001) were present. Compared with vehicle-treated rats, treatment with* A. camphorata* significantly and dose-dependently improved the neurological score at 24 h after ischemia (*P* < 0.05). In addition, compared with other blood perfusion unit (BPU) or regional cerebral blood flow (rCBF) measuring methods, laser Doppler flowmeter (LDF; Oxford Array) provides a noninvasive and continuous measure of BPU.  Figure 2(b) shows the relative changes of BPU by LDF after MCAO, 30 and 60 days of* A. camphorata* treatment alone. The results show that MCAO-induced animals sustained the most severe (*P* < 0.001) reduction in BPU. However, the low (0.25 g/kg/day) and high (0.75 g/kg/day) dose of* A. camphorata* treatment decreased the BPU compared with the vehicle, although there were no significant differences. All rats in the sham group maintained the baseline level of BPU.

### 3.3. Effects of* A. camphorata* on Subarachnoid Hemorrhage and Hemorrhagic Incidence

**Figure 3:**
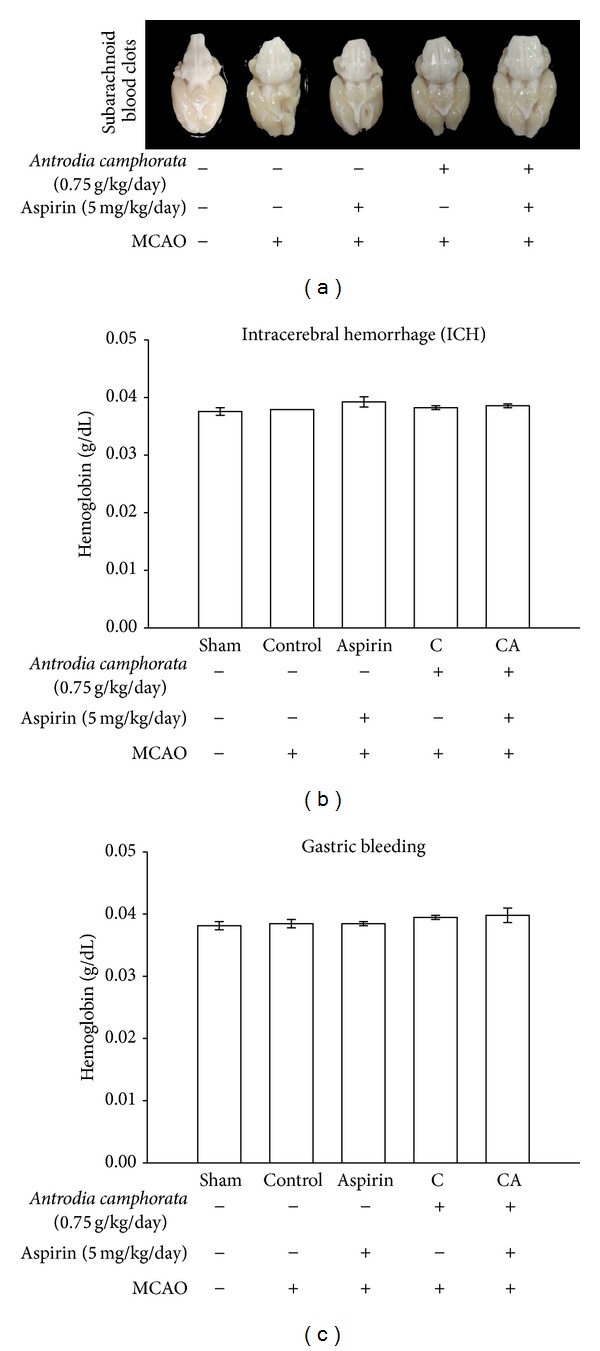
Effects of the extract of* A. camphorata* with aspirin on (a) subarachnoid hemorrhage (SAH), (b) intracerebral hemorrhage (ICH), and (c) gastric bleeding in thromboembolic stroke-induced rats. Data are presented as the means ± S.E.M. of seven similar experiments.

Representative photographs of the SAH can be seen in Figure 3(a). There is no significant difference among the groups of sham-operated, MCAO-induced, MCAO +* A. camphorata,* and MCAO +* A. camphorata* + aspirin treatment overall SAH grade. The arteries can be well recognized in the sham group. Although there is no severe effect in the SAH + MCAO group, a very little obliterated artery by the blood clot was seen. Furthermore, we found that treatment of* A. camphorata* (0.75 g/kg/day) alone or combination with aspirin (5 mg/kg/day) did not induce subarachnoid hemorrhage. In addition, the concentration of hemoglobin in the ischemic hemisphere and gastric luminal fluid in sham-operated and* A. camphorata* alone or combined with aspirin groups is almost similar (Figures 3(b) and 3(c)). Since there is no significant effect of* A. camphorata* at high dose alone or with aspirin on hemoglobin content, this treatment method is considered to be safer and does not cause any side effect. In addition, to check the intracerebral hemorrhage and gastric bleeding in studied animals hemoglobin content in the ischemic hemisphere and gastric luminal fluid was determined. The results revealed that the levels of hemoglobin were not altered in all the groups, which indicate that there are no side effects found during the treatment of* A. camphorata*, aspirin alone, or combination of both in MCAO-induced rats.

### 3.4. Effects of* A. camphorata* on Prothrombin Time (PT) and Occlusion Time (OT)

**Figure 4:**
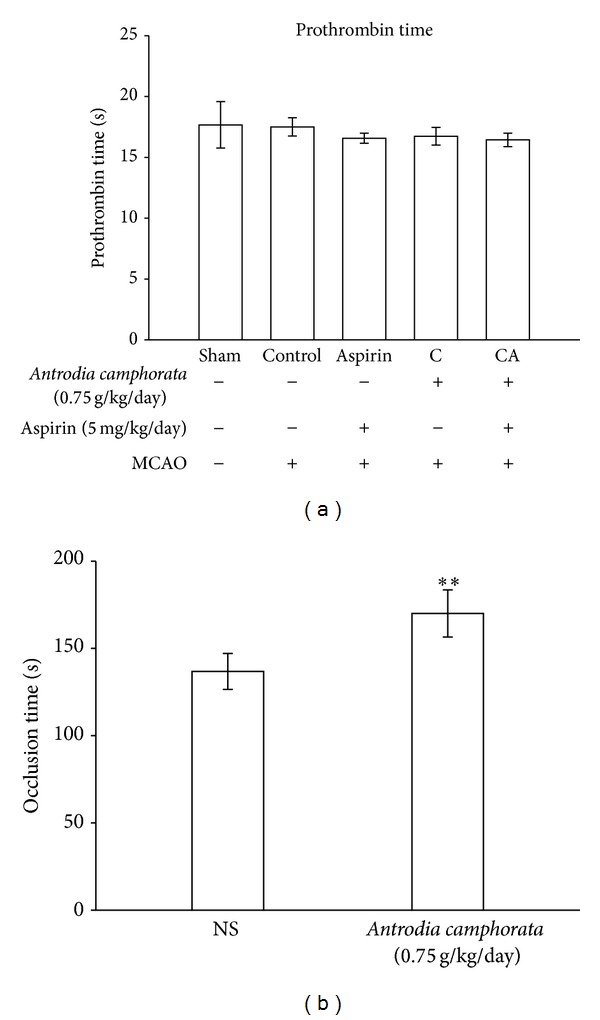
Effects of the extract of* A. camphorata* with aspirin on (a) prothrombin time (PT) and (b) occlusion time (OT) in thromboembolic stroke-induced rats. Data are presented as the means ± S.E.M. of three similar experiments.

To determine whether or not coagulation parameters are influenced by* A. camphorata* treatment alone or its combination with aspirin, PT and OT were measured in the present study.* A. camphorata* alone or with aspirin did not cause a prolongation of PT (Figure 4(a)), an observation which is at variance with a previously reported study [18]. Nevertheless, this treatment significantly (*P* < 0.01) increased prolongation of OT for inducing thrombus formation in mesenteric venules in mice when compared with fluorescein dye- (Sigma Aldrich, USA) induced untreated group (Figure 4(b)).

## 4. Discussion

There are numerous animal stroke models which were designated in the previous literature such as photochemically induced MCAO, surgical occlusion, and vessel occlusion which only simulated the aspect of vessel occlusion [18]; some aspects of human strokes can only be reproduced by these models. An ideal animal model, which resembles human embolic strokes as closely as possible, should be based on the thromboembolic occlusion of a large feeder artery. In the current study, the thromboembolic stroke model mimics human strokes more closely than do other models of cerebral ischemia [19]. Besides, animal thromboembolic strokes induced by blood clots simplify the investigation of the effects of thrombolytic therapy, which is currently the only available stroke treatment in humans. Cerebral ischemia restricted to the distribution of the thromboembolic occlusion gives rise to focal metabolic disturbances that result in infarction, neuronal necrosis, and brain edema [20]. In the present study, it is confirmed, for the first time, that oral treatment of an extract of* A. camphorata* suppresses thromboembolic stroke in rats by reducing infarct volume, improves neurological outcome, and provides neuroprotection.

Novel cellular and pharmacological therapeutic approaches are required to raise the capacity of the brain for neuroregeneration and neuroplasticity to reduce neurological deficits after stroke [21]. In the current study, we studied the neuroprotective effect of an extract of* A. camphorata* and aspirin combination treatment after an experimentally induced embolic stroke in rats. Notably, we found that administration of a combination treatment of* A. camphorata* in doses of 0.25 and 0.75 g/kg/day administered 24 h after MCAO significantly improved neurological functional outcome, reduced infarct volume, and kept sustained subarachnoid hemorrhagein rats at 60 days after MCAO. Our results also show that treatment with* A. camphorata* alone or with aspirin 24 h after MCAO resulted in no significant changes of hemorrhagic incidence, as there is no variation found in the levels of hemoglobin in cerebral hemorrhage and gastric bleeding of the ischemic hemisphere in sham,* A. camphorata* alone or in combination with aspirin groups. On the other hand,* A. camphorata* therapy alone or combined with aspirin had no effect on prothrombin time, whereas this treatment prolongs occlusion time. Together, our results demonstrate that combining* A. camphorata* with aspirin treatment has an additive effect on the treatment of this thromboembolic model of stroke.

The most commonly used tool for measuring the efficacy of neuroprotective compounds is TTC staining. Brain lesion identified by TTC staining indicates that tissues were irreversibly impaired in mitochondrial function and dehydrogenase activity [22]. Our study demonstrated that treatment with* A. camphorata* (0.25 and 0.75 g/kg/day) significantly decreased the volume of infarction following cerebral ischemia insult. It was implied that* A. camphorata* treatment could improve mitochondrial activity after brain ischemia. Behind stroke, animals consequently display a variety of neurological deficits. It is very imperative to assess neurological function outcome after stroke. The Bederson scale is a global neurological assessment that was developed to measure neurological impairments following stroke [23]. Our results revealed that* A. camphorata* alone at doses 0.25 and 0.75 g/kg/day could improve neurological behavior disturbance based on neurological deficit scores.

The duration of the ischemia and the degree of CBF or BPU reduction are the major factors to determine the severity of ischemic damage. It is generally accepted that >70% reductions in CBF are necessary to induce ischemic brain damage although some recent evidence suggests that more modest reductions may cause delayed neuronal death [24]. It was also reported that cortical injury was related to the duration of ischemia between 30 and 45 min, and beyond 37 min both magnitude and distribution of cortical injury increased markedly in a linear fashion [25]. In the present study, at 30 and 60 days MCAO induced an immediate reduction of BPU value. However, the BPU was improved in both low (0.25 mg/kg/day) and high (0.75 g/kg/day) dose groups of* A. camphorata* treatment. The increased BPU in the MCAO-induced rats may perhaps directly determine the volume of infarction. Therefore it is suggested that in our study the marked reductions of infarct volume in* A. camphorata*-treated groups may mainly result from the improved BPU in the brain during MCAO.

Increased risk of hemorrhage is a documented possible side effect of antithrombotic treatments [26]. To evaluate this aspect, the subarachnoid hemorrhage (SAH) grade was checked to know the possible side effect of* A. camphorata* alone or in combination with aspirin treatment on MCAO-induced rats. The results revealed that* A. camphorata* alone or in combination with aspirin treatment did not affect this parameter. On the other hand a literature about secondary intracerebral bleeding after stroke in rats is reported to be poor and hence bleeding rates in animals and humans may differ [27]. In this study, we focused on the efficacy of* A. camphorata* alone or with aspirin on hemoglobin content as a marker of bleeding and an important safety measure, and we found that there is no alteration in the hemoglobin level. To determine whether or not coagulation parameters and platelet functional marker are influenced by* A. camphorata* or their combination with aspirin, PT and OT were measured in the present study.* A. camphorata* or aspirin alone did not cause a prolongation of PT, an observation which is at variance with a previously reported study [17]. However,* A. camphorata* at the dose of 0.75 mg/kg/day increased the prolongation of OT. This prompts the conclusion that coagulation cascade did not part the bleeding interaction with the combination of* A. camphorata* with aspirin.

In conclusion, the present study has demonstrated that pretreatment with* Antrodia camphorata *(0.25 and 0.75 g/kg/day) is beneficial in reducing infarct volume in the focal ischemic brain injury in the embolic model.* A. camphorata* treatment is also beneficial in reestablishing blood flow to the ischemic brain by reducing perfusion deficits following ischemia and it also provides added benefits, as it did not cause any hemorrhagic incidence when used in conjunction with aspirin therapy. This evidence suggests that* A. camphorata* has neuroprotective effect against ischemic insults in our MCAO model through a mechanism of blood perfusion regulation without increasing hemorrhagic transformation.
